# The effect of neural cell integrated into 3D co-axial bioprinted BMMSC structures during osteogenesis

**DOI:** 10.1093/rb/rbab041

**Published:** 2021-08-03

**Authors:** Yi Zhang, Haiyan Chen, Xiaoyan Long, Tao Xu

**Affiliations:** 1 Precision Medicine and Healthcare Research Center, Tsinghua-Berkeley Shenzhen Institute (TBSI), Tsinghua University, Shenzhen 518055, People's Republic of China; 2 East China Institute of Digital Medical Engineering, Shangrao 334000, People's Republic of China; 3 Biomanufacturing and Rapid Forming Technology Key Laboratory of Beijing, Department of Mechanical Engineering, Tsinghua University, Beijing 100084, People’s Republic of China; 4 Key Laboratory for Advanced Materials Processing Technology, Ministry of Education, Department of Mechanical Engineering, Tsinghua University, Beijing 100084, People’s Republic of China

**Keywords:** innervation, 3D bioprinting, co-axial, BMMSC differentiation, osteogenesis

## Abstract

A three-dimensional (3D) bioprinting is a new strategy for fabricating 3D cell-laden constructs that mimic the structural and functional characteristics of various tissues and provides a similar architecture and microenvironment of the native tissue. However, there are few reported studies on the neural function properties of bioengineered bone autografts. Thus, this study was aimed at investigating the effects of neural cell integration into 3D bioprinted bone constructs. The bioprinted hydrogel constructs could maintain long-term cell survival, support cell growth for human bone marrow-derived mesenchymal stem cells (BMMSCs), reduce cell surface biomarkers of stemness, and enhance orthopedic differentiation with higher expression of osteogenesis-related genes, including osteopontin (OPN) and bone morphogenetic protein-2. More importantly, the bioprinted constructs with neural cell integration indicated higher OPN gene and secretory alkaline phosphatase levels. These results suggested that the innervation in bioprinted bone constructs can accelerate the differentiation and maturation of bone development and provide patients with an option for accelerated bone function restoration.

## Introduction

Bone defects and disorders have increased tremendously over the recent years, particularly due to trauma, infection, congenital anomalies and increased obesity, resulting in an urgent demand for bone grafts. Bone grafting is a commonly used surgical method in augmenting bone regeneration. Specifically, autologous bone grafting is considered the gold standard treatment, owing to its favorable bone quality, lower material cost, short healing time and lesser risk of disease transmission as compared to xenograft. However, the limited supply of bone autografts, risks during the collection process, and donor site complications that include the consideration of the donor site morbidity due to the loss of bone function innervated by the donor’s nerve [1], have been a continuous problem. Currently, cell-laden three-dimensional (3D) bioprinting is used as the new fabrication strategy for designing specific bone substitutes with undifferentiated stem cells, such as human mesenchymal stem cells [2] and skeletal stem cells [3], by surgeons to overcome the limitations of bone autografts. However, the success of these bone substitutes in repairing bone function is limited, owing to the lack of neural functions in these bone autografts. Researchers have previously demonstrated the integration of neural cells into the 3D bioprinted tissue to achieve a greater functional restoration of tissue regeneration [4], providing a new option for reconstructive surgery.

Cell-laden 3D bioprinted bone scaffolds provide a template for bone defect reconstruction [5, 6], while promoting proliferation, cell attachment, and restoration of vessels, muscles, nerves, and bones and offering higher stability, excellent protein factors, specific form control, and living cell gradient and types [7–9]. However, during the production of functional bone grafts, innervation should be carefully considered because it plays a pivotal role in tissue development as well as their functional control and modulation [10, 11]. Moreover, the mature development and regeneration of bone tissue relies on a robust ossification process, called the intramembranous ossification that occurs at the same time of blood vessel development and sensory neuron interaction with bone [12–14]. In addition, many researchers have reported that neurotrophins and their receptors, which are widely expressed in skeletal tissues, are key molecules in regulating the nervous system development and maintenance involved in regulating tissue formation and healing of skeletal tissues, implicated in chondrogenesis, osteoblastogenesis and osteoclastogenesis [15–17]. However, there are few reported studies on the effect of innervation during osteogenesis.

In this study, we constructed 3D cavity-like co-axial bioprinted structures to investigate the effect of neural cells on the osteogenesis of human bone marrow mesenchymal stem cells (BMMSCs). To determine the response of BMMSCs in a 3D microenvironment, we printed co-axial core-shell structures as an *in vitro* research model [18], in which the core was empty, which is similar to the marrow cavity and was filled with BMMSCs. The biomaterial shell can provide support for the 3D microenvironment. Moreover, we evaluated the effects of 3D bioprinted structures on the viability, proliferation, and differentiation of BMMSCs. Furthermore, the effects of neural cells on BMMSC differentiation based on 3D bioprinted structures were also evaluated and compared to those of the conventional two-dimensional (2D) culture.

## Materials and methods

### Cell cultures

BMMSCs were obtained from Sciencell^TM^ Research Laboratories (Carlsbad, CA, USA), and cultured and expanded to passage 5–7 in mesenchymal stem cell medium (MSCM, Sciencell, Carlsbad, CA, USA) consisted of 5% fetal bovine serum (FBS, Sciencell), 1% mesenchymal stem cell supplement (MSCGS, Sciencell) and 1% penicillin–streptomycin (Sciencell) in an incubator with an atmosphere of 37°C and 5% CO_2_. Human ReNcell VM neural progenitor cells (NSCs) were purchased from EMD Millipore (Temecula, CA, USA). The cells were labeled with green fluorescent protein (GFP) and plated onto BD Matrigel (BD Biosciences, San Jose, CA, USA)-coated T25 and T75 cell culture flasks (BD Biosciences) and expanded to passage 6–10 in DMEM/F12 (Gibco, Grand Island, NY, USA) medium supplemented with 2 μg·ml^−1^ heparin (StemCell Technologies, Vancouver, Canada), 2% (v/v) B27 neural supplement (Life Technologies, Grand Island, NY, USA), 20 ng·ml^−1^ epidemal growth factor (EGF, PeproTech, NJ, USA), 20 ng·ml^−1^ basic fibroblast growth factor (bFGF, PeproTech) and 1% (v/v) penicillin/streptomycin (Gibco) solution in a CO_2_ cell culture incubator.

For co-culture, cell suspensions containing both BMMSCs and NSCs with ratios from 1:0–500:1 were cultured on 24-well plates in MSCM consisted of 5% FBS, 1% MSCGS, 1% penicillin–streptomycin, 20 ng·ml^−1^ EGF and bFGF, incubated overnight, and then differentiated in human mesenchymal stem cell osteogenic differentiation medium (Cyagen, Guangzhou, China) for up to 14 days. The number of BMMSCs was the same with different ratios with NSCs.

### Material preparation

Two types of bioinks were applied to bioprint 3D co-axial constructs: core cell suspension bioink and shell bioink. To print cells, the optimally selected ratio of cell suspension was determined. The shell bioink was prepared by dissolving 2% alginate in 0.9% sodium chloride (NaCl, Sigma-Aldrich, Shanghai, China) solution, followed by heat sterilization. Sodium alginate was purchased from Aladdin (China). Calcium chloride (CaCl_2_, Sigma-Aldrich) was dissolved into deionized water at a final concentration of 3% (w/v) and filtered through 0.45 μm syringe filters (ThermoFisher Scientific, NY, USA). For de-crosslinking of alginate, 55 mM sodium citrate (Sigma-Aldrich) and 20 mM ethylene diamine tetraacetic acid (EDTA, Sigma-Aldrich) in 0.9% NaCl solution was prepared [19].

### Co-axial 3D bioprinting of cavity-like core-shell structures

Concentric circle structures were bioprinted with a customized computer-aided co-axial extruding bioprinting device. Firstly, the customized concentric print head with two chambers and nozzles was fabricated. Outer chamber was for shell structure, and inner chamber for core structure. Then 2% sodium alginate (w/v) dissolved in 0.9% NaCl solution (w/v) was loaded into a 10 ml syringe and printed to form shell. And cell suspension bioink was loaded into another 10 ml syringe and printed to form core. Two syringes were independently controlled by different pumps. The extrusion bioprinting speed was set as 15 ml·h^−^^1^ for shell structure and 5 ml·h ^− 1^ for core part. During bioprinting, co-axial structures were bioprinted on the receiving platform which was a tank containing 3% CaCl_2_ solution. After printing, core-shell structures were washed with 0.9% NaCl solution for three times to remove remaining calcium ions, and then placed in the growth medium overnight and then switched in the differentiation medium. The medium was changed every 3 days.

### Cell viability

Cell viability was assessed using a live/dead assay kit (KeyGEN BioTECH, Nanjing, China) according to the manufacturer’s instructions. Briefly, 8 μM propidium iodide and 2 μM Calcein-AM were mixed with 0.9% NaCl solution. The bioprinted constructs were incubated in the staining assay solution in dark at room temperature for 40–60 min. After gentle washing with 0.9% NaCl solution, live and dead cells were imaged using fluorescence microscope (Eclipse Ti2-U, Nikon, Japan). The number of live cells (green) and dead cells (red) were manually counted, and cell viability (%) was calculated (*n* = 3).

### Morphology observation

After bioprinting at 14 days, the development of cells in 3D *in vitro* core-shell structures was observed by a fluorescence microscope. And cell-laden constructs were fixed with 2.5% glutaraldehyde for 0.5 h at room temperature. The samples were then dehydrated for 20 min in a series of ethanol solutions (50%, 70%, 80%, 90%, 95%, 100%). Subsequently, the samples were lyophilized, and followed by gold spraying. The surfaces of all the structures were photographed by scanning electron microscopy (SEM, ZEISS, Germany). To observe the outcome of BMMSCs induced differentiation by differentiation medium, alizarin red staining was performed. Briefly, 2D culture cells in differentiation medium for 14 days were washed by phosphate-buffered saline (PBS). And 4% paraformaldehyde was added into well to fix these cells. The paraformaldehyde was removed, and 2D culture cells were washed with PBS. Alizarin red dye solution was added into each well for 5 min, and then removed. After washed by PBS, 2D culture cells were observed by a microscopy.

### Flow cytometry (FACS) analysis

Co-axial cell-laden structures were soaked in sodium citrate–EDTA solution to remove the alginate shell, and cells were collected to FACS analysis. A panel of antibodies conjugated to three different fluorophores was used to detect stemness of cells from 3D co-axial and 2D culture. Antibodies used were: CD73-PB450 (562430, BD Biosciences) and CD105-PE (560839, BD Biosciences). All the samples were performed by the Cytoflex (Beckman Coulter, CA, USA) and analyzed with CytExpert 2.2 software.

### Alkaline phosphatase (ALP) activity

ALP activity was quantitatively detected from the lysates of the same number of cells from 3D co-axial and 2D culture with using a commercial kit (MultiSciences Biotech, Hangzhou, China). Briefly, 50 μl lysate samples were pipetted into the wells of a 96-well plate and reacted with 50 μl staining working solution for 15 min at 37°C. Then the reaction was stopped by adding 100 μl stop buffer per well. The absorbance OD at 405 nm was measured with a microplate reader. All the ALP values were normalized according to the manufacturer’s instructions.

### Quantitative real-time reverse transcription-polymerase chain reaction (qRT-PCR)

To analyze gene expression of stemness and differentiation, qRT-PCR was performed. 3D co-axial cells were extracted by dissociation solution as described above and 2D cell were also collected. Total RNA was isolated and extracted by total RNA extraction kit (Takara, Beijing, China). RNA was reversely transcribed into cDNA with reverse transcription kit (Beyotime Biotechnology, Shanghai, China) according to the protocol. For qPCR, pairing primer sequences [20] were used (Supplementary Table S1), including alkaline phosphatase (ALP), bone morphogenetic protein-2 (BMP2), osteopontin (OPN), Runt-related transcription factor 2 (Runx-2) and glyceraldehyde 3-phosphate dehydrogenase (GAPDH). PCR amplification was performed using SYBR Green qPCR SuperMix (C11733046, Invitrogen, NY, USA) with an ABI PRISM^®^ 7500 Sequence Detection System (ThermoFisher Scientific) with the procedure of 95°C 2 min; 95°C 15 s, 60°C 30 s, reading plate, 40cycles. Each sample was repeated three times.

### Statistical analysis

Each experiment was repeated at least three times and results are presented as mean ± SD. Comparison between multiple groups was performed using two-way analysis of variance (ANOVA). Statistical significance was attained at *P *<* *0.05 (*), *P *<* *0.01 (**), *P *<* *0.001 (***), *P *<* *0.0001 (****). Comparison between two groups was performed using the paired *t*-test. Statistical significance was attained as asterisk (*). The results were analyzed and exported using GraphPad Prism 8.0 software.

## Results

### Optimal ratio of the BMMSCs to NSCs to induce bone formation

**Figure 1.**
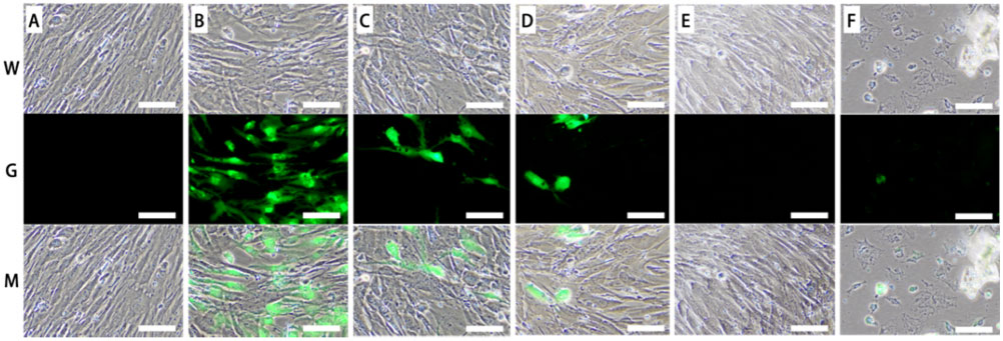
Different ratios of BMMSC-NSCs co-culture. The ratio of BMMSC to NSCs was 1:0 (**A**), 10:1 (**B**), 100:1 (**C**), 300:1 (**D**), 500:1 (**E**), 0:1 (**F**). W: white view. G: green fluorescence view for NSCs. Scale bar: 100 μm

To examine the effects of neural cells on BMMSC osteogenesis, both BMMSCs and NSCs were co-cultured in a 3D co-axial construct. It is necessary for 3D co-culture system to detect the optimized ratio of BMMSCs and NSCs to facilitate BMMSC differentiation and tissue development. Therefore, we have chosen the optimized ratio of the co-culture determined by a method performed in a 2D environment (Fig. 1). Suitable density and long-term maintenance were revaluated at different ratios of BMMSCs to NSCs in the differentiation medium for 14 days (Fig. 1). Among the following ratios, the 0:1 ratio showed poor morphology of neural cells for long-term maintenance because of no passage for NSCs. However, excessive neural cells occupied much space for BMMSC differentiation and squeezed BMMSC growth in the ratio of 10:1. Therefore, in co-culture with a 100:1 ratio, the density of both BMMSCs and neural cells might be suitable, and had the showed viability of cells under long-term maintenance (Fig. 1). These results indicated that the optimal ratio of BMMSCs to NSCs was 100:1 for suitable density and long-term maintenance.

### Bioprinting of 3 D *in vitro* co-culture constructs

**Figure 2.**
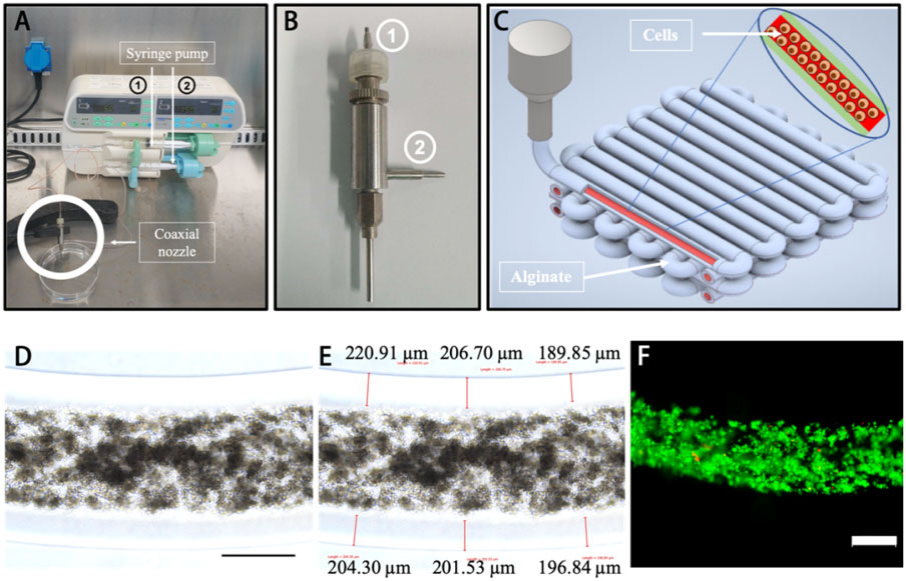
Co-axial bioprinted co-culture structure. (**A**) Co-axial bioprinting system. (**B**) The printing nozzles, consisting of &cenveo_unknown_entity_wingdings_F081; for the core part and &cenveo_unknown_entity_wingdings_F082; for the shell structure. (**C**) Design concept using 3D CAD modeling of the bioprinted construct. (**D**) Bioprinted construct (scale bar: 500 μm). (**E**) The measure results of shell thickness. (**F**) Live and dead assay of bioprinted BMMSCs after bioprinting, green for live cells, red for dead cells (scale bar: 500 μm)

Based on the 2D co-culture exploration, the 100:1 ratio of BMMSCs and NSCs was selected as the optimal ratio, and similar to the marrow cavity, 3D fiber cell-laden core-shell structures were bioprinted using a custom-made co-axial system (Fig. 2A–C). The bioprinted constructs were observed and measured with the 203.36 ± 10.47 μm shell thickness (Fig. 2D and E) which is the hydrogel diffusion limit distance for sufficient nutrient transport from the medium [21]. Moreover, the live and dead staining assay showed high cell viability in 3D co-culture constructs after printing (Fig. 2F), which demonstrates the no effect of the printing process on the cell viability. In addition, the majority of bioprinted cells in the cavity-like constructs remained viable during the 14-day culture period (Supplementary Fig. S1).

**Figure 3.**
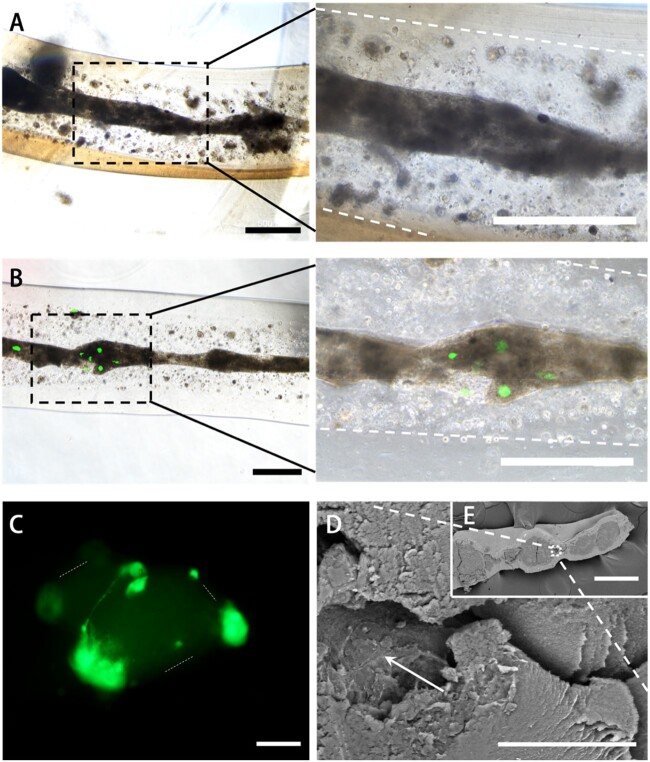
Observation of bioprinted constructs. (**A**) The BMMSC-induced growth observation images after 14 days where the boundary between the shell and core was marked out with white dotted line (scale bar: 500 μm). (**B**) The BMMSC-induced and NSCs co-culture growth observation images after 14 days in which the green fluoresce presents NSCs and the boundary of BMMSC cluster was marked out with white dotted line (scale bar: 500 μm). (**C**) NSC fluoresce observation image in 3D co-culture construct where the boundary between the shell and core was marked out with white dotted line (scale bar: 100 μm). (**D–E**) SEM images, white arrow indicated filamented cell cluster (scale bar: 30 μm for D, 300 μm for E)

Furthermore, after the 14-day culture period in the differentiation medium, we observed the morphology of *in situ* cells. In 3D bioprinted structures, BMMSCs grew and formed cell cluster fibers (Fig. 3A), and cells derived from NSCs expressed green fluoresce, and combined with cells from BMMSCs to form cell fiber in bright field view as well (Fig. 3B). In Fig. 3C, we could easily observe that cells with green fluoresce from NSCs attached the surface of the cell cluster and formed the cell filament between green cells. Moreover, cell cluster fiber could be observed and filamented cells were indicated on the surface of cell cluster in SEM images (Fig. 3D and E).

### The induced differentiation of the BMMSCs in 3D bioprinted constructs

**Figure 4.**
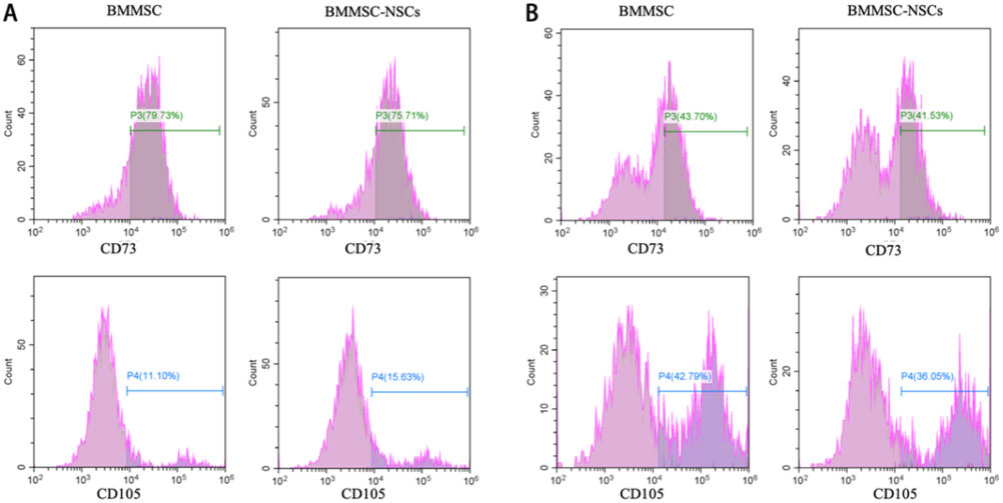
BMMSC differentiation. (**A**) CD73 and CD105 biomarker detection of cells from 2D culture. (**B**) CD73 and CD105 biomarker detection of cells from 3D bioprinted constructs

After the 14-day culture period, the differentiated cells were harvested from 3D bioprinted structures and analyzed using a flow cytometer. The expression of CD73 and CD105 in BMMSCs decreased (< 80%) and showed differentiation (Fig. 4). Moreover, CD73 of the harvested cells in the 3D bioprinted microenvironment was lower (42.62 ± 1.53) compared than the CD73 in the 2 D culture (77.72 ± 2.84) (Fig. 4). However, the positive proportion of CD105 of cells in 3D constructs was higher (39.42 ± 4.77) than that in 2D culture (13.37 ± 3.20) (Fig. 4). These results showed the different characteristic changes of BMMSCs in 3D and 2D microenvironments.

Meanwhile, in 3D bioprinted constructs, CD73 (41.53%) and CD105 (36.05%) in the co-culture of BMMSCs and NSCs showed a relatively lower expression than the expression of CD73 (43.70%) and CD105 (42.79%) in the pure BMMSC culture (Fig. 4B). The results indicated that neural cells affected the stem cell differentiation.

### The effect of neural cells on the bone-related gene expression in 3D bioprinted constructs

**Figure 5.**
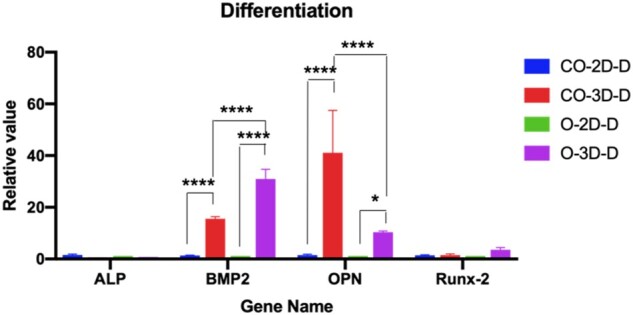
Osteogenesis analysis by gene expression. CO-2D-D: 2D co-culture, CO-3D-D: 3D co-culture, O-2D-D: 2D only BMMSC culture, O-3D-D: 3D only BMMSC culture. Statistical significance was attained at *P <* *0.05, **P <* *0.01, ***P <* *0.001, ****P <* *0.0001

The bone-related gene expression analysis was performed using specific primers to further understand the effects of neural cells on the development of bone tissues (Supplementary Table S1) using qRT-PCR. The cells cultured in the differentiation medium for 14 days demonstrated the formation of calcium salts in mineralized nodules of osteoblasts through alizarin red staining assay (Supplementary Fig. S2) and were used for qRT-PCR assays. The results showed that expression of OPN and BMP2 genes was significantly higher in 3D bioprinted constructs than in 2D culture (*P *<* *0.05, two-way ANOVA), either for co-culture of BMMSCs and NSCs or pure BMMSCs (Fig. 5). We demonstrated that 3D bioprinted constructs are necessary in long-time bone-induced developments by providing a more suitable microenvironment than 2D bioprinted constructs. Moreover, in the 3D bioprinted groups, the two genes have exhibited significantly different expressions between the 3D co-culture and pure cell culture microenvironment. Meanwhile, OPN levels were higher in the 3D co-culture than in the 3D BMMSC-only culture. On the other hand, however, the BMP2 expression was higher in the 3D BMMSC-only culture than in the 3D co-culture (Fig. 5). These results indicate that neural cells have a complex effect on bone-induced differentiation processes.

### Increased ALP with neural cells in 3D co-culture bioprinted constructs

**Figure 6.**
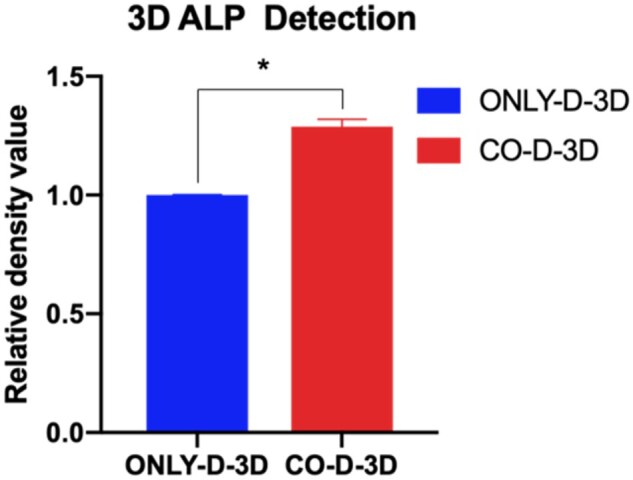
ALP activity of BMMSC in bioprinted constructs. ONLY-D-3D: 3D BMMSC-only culture, CO-D-3D: 3D co-culture. Asterisk (*) presents the significant difference (*P *<* *0.05, paired *t*-test)

The effect of neural cells on osteogenesis was evaluated by the ALP assay for cells in a 3D co-culture bioprinted system with the ability to provide long-term cell viability and a bone-induced microenvironment. Similar number of cells was extracted from 3D bioprinted constructs and broken using the ultrasonic cell disruption system, and ALP levels were assessed using an ALP assay kit. The results showed that ALP levels were significantly higher in the 3D co-culture system than in the BMMSC culture system (Fig. 6, *P *<* *0.05, paired *t*-test), suggesting that neural cells induced osteogenic processes in BMMSCs.

## Discussion

As sensory and sympathetic neurons are well known to innervate the marrow cavity, trabecular bone and periosteum of long bones [22], it is difficult to observe the neural regulation of bone remodeling and healing. This study investigated the effect of neural cells on the BMMSC differentiation in a 3D cavity-like microenvironment. We have chosen the suitable and optimized ratio of cells for 2 weeks using a 2D co-culture system for further 3D bioprinting experiments, and we found that 100:1 BMMSCs to NSCs was the optimal ratio that showed suitable density and long-term active and morphological maintenance (Fig. 1).

Since most bone defects are reconstructed with patient-customized grafts to restore structure and function [23], therapeutic strategies for specific bone defect repair are limited. 3D bioprinted tissue constructs with bioengineered structural and bioactive functional features of *in vivo* bone tissue have provided a promising option for bone-injured patients to recover bone function. Recent advances in orthopedic 3D bioprinting have enabled the establishment of suitable scaffolds for improving cell viability, proliferation and homing, osteogenic differentiation, vascularization, host integration, and load bearing [24, 25]. In this study, we biofabricated co-axial cavity-like core-shell human bone tissue structures by integrating neural cell components in 3D bioprinted constructs (Fig. 2). Most of the cells in bioprinted constructs remained alive for at least 14 days (Fig. 2F and Supplementary Fig. S1), and cells gathered to form cell fibers (Fig. 3A, B and E). This result indicates that a 3D bioprinted environment can maintain cell viability and support long-term cell proliferation [26].

*In vivo* bone regeneration requires stem cell (BMMSCs) migration and bone formation [27]. Our bioprinted cavity-like core-shell constructs had the core space for cell migration and formed cell groups, representing cell fibers and the close contact of neural cells and BMMSCs in our results (Fig. 3). These cell fibers easily produce the extracellular matrix (ECM), remodeling complete bone healing [28]. Our results indicated that 3D bioprinted cavity-like constructs might promote the accelerated differentiation and maturation of BMMSCs into osteogenesis *in vitro* (Figs 4 and 5). Therefore, it was necessary to consider the integration of the whole structure and function when 3D bioprinted biomimetic bone constructs are biofabricated [29].

For the functional restoration and healing of injured bone tissue *in vivo*, indispensable innervation of the host nerve is critical [11]. Unfortunately, there is no strategy for exploring and researching the innervation of bone healing *in vivo* with relevant *in vitro* models that have not been fully developed. Several 3D bioprinted co-culture scaffolds with BMMSCs and neurons have been reported, but these studies did not focus on the effect of neural cells on BMMSC differentiation for bone healing [2, 30]. In this study, we fabricated co-axial core-shell constructs that benefited the differentiation tendency of cell fibers [31], and our results demonstrated that neural cell integration into 3D bioprinted co-axial BMMSC-laden constructs influenced the differentiation and survival of BMMSCs (Fig. 5), and further improved the ALP level for osteogenesis *in vitro* (Fig. 6). It is also noteworthy that although the OPN gene showed higher expression in 3D co-culture than in 3D BMMSC-only culture, BMP2 expression was lower in 3D co-culture constructs than in 3D BMMSC-only culture systems. It was assumed that the addition of neural cells influenced the total RNA of cells in 3D co-culture systems, and then reduced the real RNA from pure BMMSCs for RT-PCR assays. Thus, it is possible that the real level of BMP2 genes in 3D co-culture constructs would be underestimated.

The neurotrophic factors that were released from neural cells was reported to have the ability to regulate bone healing development [15], and several studies have shown the role of neurotrophic factors in stromal cell osteogenic differentiation [32, 33]. The present study, which included and considered the innervation in bone tissues, is promising for the development of new therapeutic options for bone grafts. However, further research is needed for *in situ* observation and control of function and regulation of the sensory and sympathetic neurons in bone tissue constructs. For example, the different frequent electrical stimulus may be added and used to stimulate neural cells, and the behavior of BMMSCs could be observed *in situ*.

## Conclusion

Core-shell constructs encapsulating BMMSCs integrated with neural cells were successfully bioprinted using a co-axial bioprinting platform. We have demonstrated that the printed hydrogel constructs can support long-term cell survival and growth. Moreover, bioprinted BMMSCs reduced the cell surface biomarkers of the stemness and enhanced the orthopedic differentiation. Expression of osteogenesis-related genes, including OPN and BMP2, in 3D bioprinted constructs was higher than that in 2D culture cells. Moreover, based on the results of gene qRT-PCR and ALP assays in this study, innervation could improve the differentiation and maturation of bone development based on an *in vitro* model. With further advances, the integration of neural cells into bioengineered bone constructs pose as an effective therapeutic approach for repairing extensive bone defect injuries with accelerated functional restoration capacity.

## Supplementary data

Supplementary data are available at *REGBIO* online.

## Supplementary Material

rbab041_Supplementary_Data
