# Supplementary material for: The effect of neural cell integrated into 3D co-axial bioprinted BMMSC structures during osteogenesis
Source: Regen Biomater. 2021 Aug 3;8(5):rbab041. doi: 10.1093/rb/rbab041 (PMC8329473; doi:10.1093/rb/rbab041)
Supplement: rbab041_Supplementary_Data [file rbab041_supplementary_data.zip › Supplementary Data with revision marks.docx]

Supplementary Data

**The effect of neural cell integrated into 3D co-axial bioprinted BMMSC structures during osteogenesis**

**Yi Zhang^1^, Haiyan Chen^1^, Xiaoyan Long^2^, Tao Xu ^1,3,4 *^**


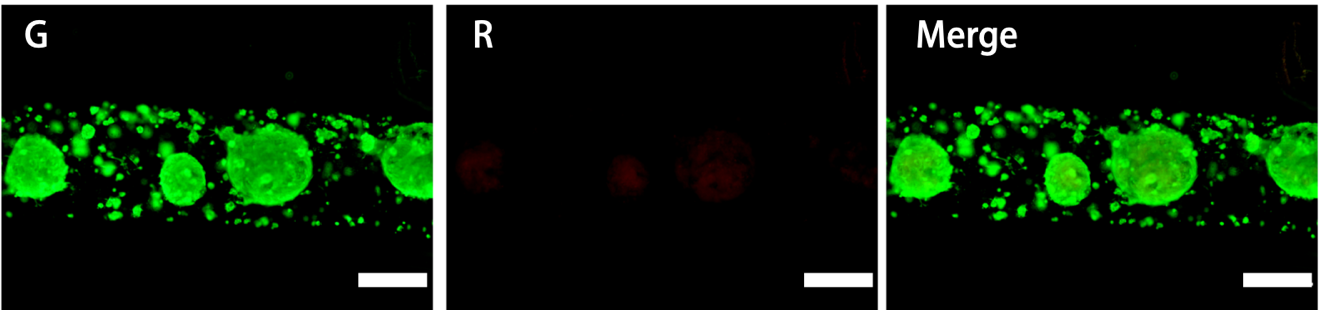


**Figure S1.** Live/dead staining assay for BMMSCs after 14-day culture. G: green fluorescence for live cells, R: red fluorescence for dead cells. Scale bar: 500 μm.


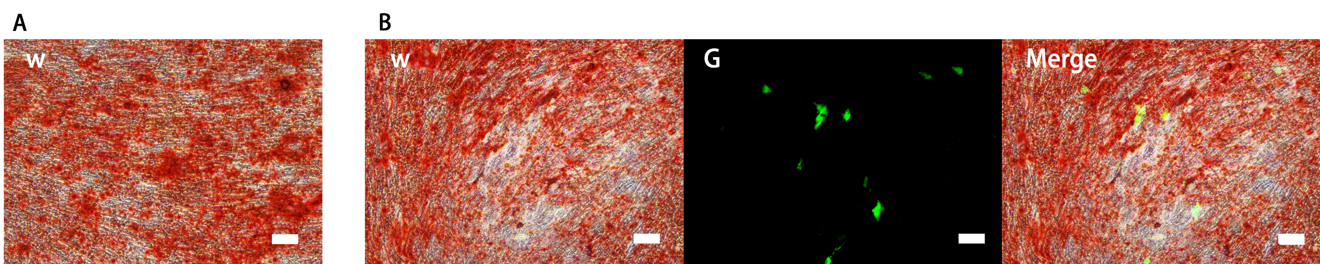


**Figure S2.** Alizarin red staining assay after 14-day co-culture under differentiation medium. W: white view, G: green fluorescence for NSCs. Scale bar: 100 μm.

**Table S1.** Specific pairing primer sequences.

| **Gene** | **Sequence (5′-3′)** |
| --- | --- |
| Alp-Forward | 5′-ACCATTCCCACGTCTTCACATTT-3′ |
| Alp-Reverse | 5′-AGACATTCTCTCGTTCACCGCC-3′ |
| bmp2-Forward | 5′-GCCCTTTTCCTCTGGCTGAT-3′ |
| bmp2-Reverse | 5′-TTGACCAACGTCTGAACAATGG-3′ |
| OPN-Forward | 5′-ATGAGATTGGCAGTGATT-3′ |
| OPN-Reverse | 5′-TTCAATCAGAAACCTGGAA-3′ |
| Runx2-Forward | 5′-AGATGATGACACTGCCACCTCTG-3′ |
| Runx2-Reverse | 5′-GGGATGAAATGCTTGGGAACT-3′ |
| GAPDH-Forward | 5′-GGGCATGAACCATGAGAAGT-3′ |
| GAPDH-Reverse | 5′-GTCTTCTGGGTGGCAGTGAT-3′ |
