# Supplementary material for: The effect of neural cell integrated into 3D co-axial bioprinted BMMSC structures during osteogenesis
Source: Regen Biomater. 2021 Aug 3;8(5):rbab041. doi: 10.1093/rb/rbab041 (PMC8329473; doi:10.1093/rb/rbab041)
Supplement: rbab041_Supplementary_Data [file rbab041_supplementary_data.zip › Point-by-point response to the reviewers’ comments-.docx]

**Point-by-point response to the reviewers’ comments**

Jun 8^th^, 2021

Dear Dr. Nicholas Peppas and dear Reviewers,

Thank you for giving us the opportunity to submit a revised draft of the manuscript titled "The effect of neural cell integrated into 3D co-axial bioprinted BMMSC structures during osteogenesis". We appreciate the time and effort that you and the reviewers have dedicated to providing your constructive comments on our manuscript. We have been able to incorporate changes to reflect the insightful suggestions provided by the reviewers. A detailed point-by-point response to the reviewers’ comments is listed below. New text passages are written in blue color.

We are very grateful for all your comments and for publication of this paper. If you have any questions, we shall be at your disposal.

Sincerely,

Tao Xu, PhD, Professor

Biomanufacturing Center,

Department of Mechanical Engineering,

Tsinghua University,

Beijing, 100084, China

TEL: +86 18210596832

E-mail: taoxu@tsinghua.edu.cn

**Comments from** **Reviewer #1**

Comments to the Author

In the manuscript, the authors investigated the effects of neural cells on the osteogenesis of bioprinted 3D BMMSCs-laden bone constructs after integration into them, and confirmed the positive role of neural cells by the in vitro cell culture experiments. The topic is interesting. However, there are some issues need be addressed.

**Reviewer #1-1.** During the coaxial bioprinting, two types of bioinks were used. What’s the core cell suspension bioink? Only cell suspension? If so, how it was gelled or cured to make both ends of the 3D constructs sealed?

**Response to Reviewer #1-1.**

Thank you for your comment. Yes, cell suspension is in core part. Actually, both ends of the 3D constructs could be sealed by controlling the extrusion time of the shell of alginate before or after that of core part. At the beginning of the bioprinting, the extrusion time of the alginate shell was ahead of core part so that the beginning end of the constructs could be sealed by the crosslinking of excess outer alginate shell with 3% CaCl_2_. Similarly, at the ending of the printing procedure, the extrusion end time of the alginate shell was later than that of core part so that the end of the constructs could be sealed by the crosslinking of excess outer alginate shell.

**Reviewer #1-2.** In Fig. 1, what’s the criterion to judge the best one is 100:1 ratio? In fact, more live NSCs were found in the group of 10:1. Besides, in the group of 0:1, almost no live cells could be observed, why?

**Response to Reviewer #1-2.**

Thank you for pointing this out. The criterion to judge the suitable ratio of BMMSC to NSCs is based on the suitable density and long-term maintenance. Although more live NSCs were found in the ratio 10:1, excessive neural cells occupied much space for BMMSC differentiation and squeezed BMMSC growth. Actually, neural cells in *in vivo* various tissues also have suitable density. For example, a high neural cell component to skeletal muscle cells is known to damage the muscle tissue regeneration by interrupting the cell fusion and decreasing myotube formations [1]. Moreover, in our study, the suitable density resulting from the suitable ratio of 100:1 could be observed in Fig. 1 in our manuscript and provided the suitable condition and results for the further study of the effect of neural cell during osteogenesis.

Moreover, in the group of 0:1, almost no live cells could be observed, because in the group of 0:1, no passage for NSCs for 14-day differentiation culture could result in cell contact inhibition and cell death and apoptosis. Therefore, the ratio of 0:1 is not suitable. We have added above information as follows:

**Page 7, lines 183–186: “***Among the following ratios, the 0:1 ratio showed poor morphology of neural cells for long-term maintenance because of no passage for NSCs. However, excessive neural cells occupied much space for BMMSC differentiation and squeezed BMMSC growth in the ratio of 10:1.***”**

**Reviewer #1-3.** Where are the SEM images of the lyophilized constructs? What’s the size of the core and the shell? The structure of the shell and the scaffolds should be given to verify its ability of nutrients transmission for inner cells growth.

**Response to Reviewer #1-3.**

Thanks for your advice. We have provided the SEM images in Fig. 3 as 3D and 3E. According to your suggestion, we have measured the shell thickness (203.36 ± 10.47 μm) as shown in Fig. 2E and added the descriptions about the structure of the shell and the scaffolds in lines 199–201 in our manuscript. The shell thickness was approximately 200 μm, which is the hydrogel diffusion limit distance [2]; therefore, the integrated bioprinted constructs were theoretically allowed to provide sufficient nutrient transport from the medium. We have revised this part as follows:

**Page 8, lines 199–201:** “*The bioprinted constructs were observed and measured with the 203.36 ± 10.47 μm shell thickness (Fig. 2D and E) which is the hydrogel diffusion limit distance for sufficient nutrient transport from the medium [21].*”

**Reviewer #1-4.** In Fig. 2D-G, some information were missing. For example, what’s the kinds of cells and culture time in Fig. 2D? How to confirm the cell fibers formed by BMMSCs, and NSCs contact with them?

**Response to Reviewer #1-4.**

Thank you for your advice. We have revised our Fig. 2D as Fig. 2F and added the information about the kinds of cells and culture time in Fig. 2F in line 209 of our manuscript.

**Page 9, line 209:** “*Live and dead assay of bioprinted BMMSCs after bioprinting.*”

We have revised our Fig. 2E and 2F as Fig. 3A and 3B and marked out boundary between the shell and core with white dotted line in Fig. 3A and 3B in our manuscript to demonstrate the cell fiber in cell suspension core part. In Fig.3B, cells derived from NSCs expressed green fluoresce, and cells from BMMSCs formed cell fiber in bright field view. Therefore, we have also found the image evidence of the interaction between NSCs and BMMSCs in Fig.3C in our revised manuscript. In our manuscript, we have revised Fig. 2F as Fig.3C, and added white dotted line to indicate the boundary of BMMSC cluster in Fig.3C where we could easily observe that cells with green fluoresce (NSCs) attached to the surface of the cell cluster and formed the cell filament between green cells. And we have added the above description in line 212–217 in our manuscript as follows:

**Page 9, lines 212–217:** “*In 3D bioprinted structures, BMMSCs grew and formed cell cluster fibers (Fig. 3A), and cells derived from NSCs expressed green fluoresce, and combined with cells from BMMSCs to form cell fiber in bright field view as well (Fig. 3B). In Fig. 3C, we could easily observe that cells with green fluoresce from NSCs attached the surface of the cell cluster and formed the cell filament between green cells. Moreover, cell cluster fiber could be observed and filamented cells were indicated on the surface of cell cluster in SEM images (Fig. 3D and E).*”

**Reviewer #1-5.** According to the current study, the 2D culture was performed in the 24-well plates with no scaffold materials, and in the 3D constructs, the cells were seeded on the inner surface of the shell. In fact, the cells concentrated in the core of the constructs. Can this be regarded as 3D culture? Is the comparison between 2D and 3D meaningful? Moreover, during PCR and ALP analysis, the lysates came from the mixture of both cells, can the up-regulated ALP activity or gene expressions be only ascribed to the BMMSCs?

**Response to Reviewer #1-5.**

Thank you for pointing this out. Co-axial 3D bioprinting has been a mature technology in tissue engineering, especially for fibre-shaped functional tissues that mimic muscle fibres, blood vessels or nerve networks *in vivo* [3]. Cell suspension as core bioink for 3D bioprinting is very meaningful based on previous work of our lab. It is found that in glioma study, coaxial extrusion bioprinted glioma cell hydrogel microfiber where U118 cell suspension is as core bioink, is a preferable platform for mimicking glioma microenvironment, as well as for drug development and screening [4]. Moreover, 3D co-axial extruded core-shell microenvironment has an important role in core cell growth and different effect on the behavior of mouse neural progenitor cell (NE-4C) compared to extrusion-based bioprinted cell-laden structures [5].

Meanwhile, during PCR and ALP analysis, we have compared the ALP activity or gene expressions of 3D co-culture system containing NSCs and BMMSCs with that of 3D only BMMSC culture system. Our results showed up-regulated ALP activity or gene expressions in 3D co-culture system, suggesting that neural cells could induce osteogenic processes in BMMSCs. Actually, above conclusions were based on the following reasons. Firstly, we have set the same cell number in ALP analysis and the same RNA quantity in PCR analysis for 3D co-culture system containing NSCs and BMMSCs and 3D only BMMSC culture system. And we have found that BMMSCs under differentiation medium for 14 days could differentiated into osteoblasts (Fig. S2). As we all know, both ALP protein and OPN gene are biomarkers for osteogenesis and have higher expression in BMMSC-induced cells than other kinds of cells [6-8]. So, it is clear that pure BMMSC-induced osteogenic cells can produce more ALP protein and OPN gene than the same number of BMMSC-induced osteogenic cells and NSCs or the same RNA quantity from BMMSC-induced osteogenic cells and NSCs. Therefore, up-regulated results are reasonable. Maybe, the up-regulated level may be higher than current results.

**Reviewer #1-6.** The main text only have five figures. So, it’s better to move the supplementary figures to the main text, and Fig. 1 should be reorganized to make the reader understand the cell state more clearly.

**Response to** **Reviewer #1-6.**

Thank you for your advice. We have added more representative figures in our manuscript and six figures have now been in our revised manuscript. And we have revised our Fig.1 with using clear figures with large multiply to allow the readers to understand the cell states in our manuscript.

**Reviewer #1-7.** In Fig. 4, Alp and bmp2 should be replaced with ALP and BMP2.

**Response to** **Reviewer #1-7.**

Thanks for your suggestion. We have replaced Alp and bmp2 with ALP and BMP2 to in our revised manuscript.

**Reviewer #2:**

Comments to the Author

This manuscript introduced a core-shell construct encapsulating BMMSCs integrated with neural cells to investigate the effects of neural cell integration into 3D bioprinted bone constructs.  The proposed construct was achieved by a co-axial bioprinting platform.  The printed hydrogel constructs can support long-term cell survival and growth.  The neural cell integration indicated higher OPN gene and secretory alkaline phosphatase levels, suggesting that the innervation in bioprinted bone constructs can accelerate the differentiation and maturation of bone development.

The concept of this MS is very innovative. However, there are some issues need to be addressed. Authors may see the comments as follow:

**Reviewer #2-1.** There is no mechanical properties characterization of the structure obtained by 3D printing in the article, because the article mentioned that in-situ repair will be attempted in the future, so mechanical characterization is necessary.

**Response to Reviewer #2-1.**

Thank you for your advice. We think that our study has some limitation in *in situ* observation and control of function and regulation of the sensory and sympathetic neurons in bone tissue constructs. In future work, we plan to use different frequent electrical stimulus to stimulate neural cells and observe the behavior of BMMSCs *in situ*. Maybe, our content didn’t express our concern clearly. And we have revised our manuscript in lines 323–325 as follows:

**Page 15, lines 323–325:** *“For example, the different frequent electrical stimulus may be added and used to stimulate neural cells, and the behavior of BMMSCs could be observed in situ.”*

**Reviewer #2-2.** Bone repair is a relatively slow process. Most of the existing studies have a research duration of about 28 days, and a small number of studies have a duration of about 21 days, while the cell culture time of this research is only 14 days. Are there any special considerations for choosing such a short culture time?

**Response to** **Reviewer #2-2.**

Thank you for this comment. Because we have observed that the cells cultured in the differentiation medium for 14 days demonstrated the formation of calcium salts in mineralized nodules of osteoblasts (Fig. S2). So, 14 days should be suitable for osteogenesis in our study.

**Reviewer #2-3.** Page 2, Line 3-4: “owing to its favorable bone quality, lower material cost, short healing time, and lesser risk of disease transmission.” The author should state the object of comparison here.

**Response to Reviewer #2-3.**

Thank you for suggestion. We have added the xenograft as the object of comparison and revised our manuscript in line 49 in our manuscript as follows:

**Page 2, lines 43–44:** *“owing to its favorable bone quality, lower material cost, short healing time, and lesser risk of disease transmission as compared to xenograft.”*

**Reviewer #2-4.** Page 3, Line 23-25. For the co-culture medium, the author supplemented FBS, MSCGS, EGF, and bFGF, but without B27 neural supplement.  Did this affect the function of the NSCs?

**Response to** **Reviewer #2-4.**

Thank you for pointing this out. We have tested the effect of co-culture medium on NSCs during exploration and found the good morphology for one day culture in the following Fig. 1. So, this may not affect the growth of NSCs.

**Figure 1.** Observation under co-culture medium for 1 day. Scale bar: 100 μm.

Moreover, compared to B27 supplement, FBS has more abundant nutrient and growth factors including serum component which could induce stem cell differentiation [9]. And during osteogenesis, it was beneficial for BMMSCs that NSCs were induced into neuron.

**Reviewer #2-5.** Page 4, Section 2.4. The author mentioned fluorescence microscope and SEM.  The results of the fluorescence microscope are in Fig 1 and 2.  I would like to make sure which part is the result of SEM.

**Response to** **Reviewer #2-5.**

Thank you for pointing this out. We have added the SEM images in Fig.3D and 3E in our revised manuscript.

**Reviewer #2-6.** Figure 1. The author optimized a suitable ratio of BMMSCs and NSCs to induce bone formation. How to define suitable density and long-term maintenance? Are there any data or parameters to prove the suitable density and maintenance?

**Response to** **Reviewer #2-6.**

Thank you for your advice. The criterion to judge the suitable ratio of BMMSC to NSCs is based on the suitable density and long-term maintenance. Although more live NSCs were found in the ratio 10:1, excessive neural cells occupied much space for BMMSC differentiation and squeezed BMMSC growth. Actually, neural cells in *in vivo* various tissues also have suitable density. For example, a high neural cell component to skeletal muscle cells is known to damage muscle tissue regeneration by interrupting the cell fusion and decreasing myotube formations [1]. Moreover, in our study, the suitable density resulting from the suitable ratio of 100:1 could be observed in Fig. 1 in our manuscript and may provide the suitable condition and results for the further study of the effect of neural cell during osteogenesis.

**Reviewer #2-7.** Figure 2. The obtained construct in Fig 2c was a multilayer structure, which differs from the filamentous structure observed in Fig 2E and F.

**Response to** **Reviewer #2-7.**

Thank you for pointing this out. In this study, we aimed to explore the effect of neural cell integrated into 3D co-axial bioprinted BMMSC structures during osteogenesis. Therefore, the fiber-shaped 3D core-shell constructs were suitable for the characterization, such as microscope observation, live & dead assay and IF analysis. So, we focused on single fiber-shaped 3D core-shell constructs of multilayer scaffold for primary assays. At the same time, we have revised our Fig. 2E and 2F as Fig. 3A and 3B and marked out boundary between the shell and core with white dotted line in Fig. 3A and 3B in our manuscript to demonstrate core-shell structure.

**Reviewer #2-8.** Page 11, Line 6-8: “Our results showed that 3D bioprinted cavity-like constructs promoted the accelerated differentiation and maturation of BMMSCs into osteogenesis in vitro.”  Fig 3 and Fig 4 indicated the effect of 3D structure as well as NSCs on BMMSCs.  However, the influence of the cavity-like structure needs further comparison with gel-bedding structure.

**Response to** **Reviewer #2-8.**

Thank you for pointing this out. Good advice. This content about the influence of the cavity-like structure will be studied in our futhure work. To clarify the suitable expression for our current work, we have changed the word “showed” to “indicated” and revised our content in lines 299**–**301 in our manuscript as follows:

**Page 14, lines 299–301:** *“Our results indicated that 3D bioprinted cavity-like constructs might promote the accelerated differentiation and maturation of BMMSCs into osteogenesis in vitro (Fig. 4 and 5).”*

**Minor:**

**Reviewer #Minor-1.** The manuscript needs a thorough language check (like sodium chloride and NaCl)

**Response to Reviewer #Minor-1.**

Thank you for your advice. We have revised our manuscript again for the language check. Please review our new manuscript.

**Reviewer #Minor-2.** Please re-check the description of reagent suppliers.

**Response to Reviewer #Minor-2.**

Thanks for your advice. We have checked and revised the description of reagent suppliers in Materials and Methods section in our manuscript.

**Reviewer #Minor-3.** Figure 2D, E, F: It is recommended to mark out the core and shell.

**Response to Reviewer #Minor-3.**

Thanks for your advice. We have revised our Fig. 2E and 2F as Fig.3A and 3B and marked out the boundary between the core and shell. And We have revised our Fig. 2D as Fig. 2F, and added new Fig. 2D and 2E, therefore, it was obvious that fluorocytes in Fig. 2F occupied the core.

**Reviewer #Minor-4.** Inconsistent line spacing and font size in the Material and Methods section.

**Response to Reviewer #Minor-4.**

Thank you for pointing this out. We have revised the content and uniformed the line spacing and font size in our manuscript.

**Reviewer #3:** Review Editor

Comments to the Author:

Please consider the comments of the reviewers and provide a drastically improved document.

**Response to Review Editor.**

Thank you for your advice. With the help of the reviewers, we have revised our manuscript carefully and improved our document based on the above comments of two reviewers. Now new revised manuscript has already been prepared carefully.

**References**

1.	Hinds, S.; Tyhovych, N.; Sistrunk, C.*, et al.* Improved Tissue Culture Conditions for Engineered Skeletal Muscle Sheets. *The Scientific World Journal* 2013; **2013**: 370151.

2.	Zhang, Y.; Yu, Y.; Chen, H.*, et al.* Characterization of printable cellular micro-fluidic channels for tissue engineering. *Biofabrication* 2013; **5**: 025004.

3.	Onoe, H.; Okitsu, T.; Itou, A.*, et al.* Metre-long cell-laden microfibres exhibit tissue morphologies and functions. *Nature Materials* 2013; **12**: 584-90.

4.	Wang, X.; Li, X.; Dai, X.*, et al.* Coaxial extrusion bioprinted shell-core hydrogel microfibers mimic glioma microenvironment and enhance the drug resistance of cancer cells. *Colloids Surf B Biointerfaces* 2018; **171**: 291-9.

5.	Li, X.; Wang, X.; Chen, H.*, et al.* A comparative study of the behavior of neural progenitor cells in extrusion-based in vitro hydrogel models. *Biomedical Materials* 2019; **14**: 065001.

6.	Chen, J.K.; Shapiro, H.S.; Sodek, J. DEVELOPMENTAL EXPRESSION OF BONE SIALOPROTEIN MESSENGER-RNA IN RAT MINERALIZED CONNECTIVE TISSUES. *Journal of Bone and Mineral Research* 1992; **7**: 987-97.

7.	Wu, C.T.; Zhou, Y.H.; Xu, M.C.*, et al.* Copper-containing mesoporous bioactive glass scaffolds with multifunctional properties of angiogenesis capacity, osteostimulation and antibacterial activity. *Biomaterials* 2013; **34**: 422-33.

8.	Ojansivu, M.; Rashad, A.; Ahlinder, A.*, et al.* Wood-based nanocellulose and bioactive glass modified gelatin–alginate bioinks for 3D bioprinting of bone cells. *Biofabrication* 2019; **11**: 035010.

9.	Yoo, D.H.; Im, Y.S.; Jo, E.H.*, et al.* Simple differentiation method using FBS identifies DUSP6 as a marker for fine-tuning of FGF-ERK signaling activity in human pluripotent stem cells. *Biochemical and Biophysical Research Communications* 2020; **521**: 375-82.
